# Relation between body composition and severe diarrhea in patients treated with preoperative chemoradiation with capecitabine for rectal cancer: a single-centre cohort study

**DOI:** 10.1186/s12876-021-01886-3

**Published:** 2021-08-04

**Authors:** J. M. van Rees, W. Hartman, J. J. M. E. Nuyttens, E. Oomen-de Hoop, J. L. A. van Vugt, J. Rothbarth, C. Verhoef, E. van Meerten

**Affiliations:** 1 Department of Surgical Oncology and Gastrointestinal Surgery, Erasmus MC Cancer Institute, Rotterdam, The Netherlands; 2 Department of Medical Oncology, Erasmus MC Cancer Institute, Rotterdam, The Netherlands; 3 Department of Radiation Oncology, Erasmus MC Cancer Institute, Rotterdam, The Netherlands

**Keywords:** Rectal cancer, Chemoradiation therapy, Adverse events, Body composition, Skeletal muscle mass

## Abstract

**Background:**

Chemoradiation with capecitabine followed by surgery is standard care for locally advanced rectal cancer (LARC). Severe diarrhea is considered a dose-limiting toxicity of adding capecitabine to radiation therapy. The aim of this study was to describe the risk factors and the impact of body composition on severe diarrhea in patients with LARC during preoperative chemoradiation with capecitabine.

**Methods:**

A single centre retrospective cohort study was conducted in a tertiary referral centre. All patients treated with preoperative chemoradiation with capecitabine for LARC from 2009 to 2015 were included. Patients with locally recurrent rectal cancer who received chemoradiation for the first time were included as well. Logistic regression analyses were performed to identify risk factors for severe diarrhea.

**Results:**

A total of 746 patients were included. Median age was 64 years (interquartile range 57–71) and 477 patients (64%) were male. All patients received a radiation dosage of 25 × 2 Gy during a period of five weeks with either concomitant capecitabine administered on radiation days or continuously during radiotherapy. In this cohort 70 patients (9%) developed severe diarrhea. In multivariable logistic regression analyses female sex (OR: 4.42, 95% CI 2.54–7.91) and age ≥ 65 (OR: 3.25, 95% CI 1.85–5.87) were the only risk factors for severe diarrhea.

**Conclusions:**

Female patients and patients aged sixty-five or older had an increased risk of developing severe diarrhea during preoperative chemoradiation therapy with capecitabine. No relation was found between body composition and severe diarrhea.

## Background

With approximately 4.500 of newly diagnosed cases per year in the Netherlands alone, rectal cancer is a common malignancy for both male and female [1]. Management of rectal cancer has rapidly changed due to the advent of new multimodality treatment modalities and has led to major improvements in oncologic outcomes [2–4].

The golden standard for curative treatment of rectal cancer still consists of surgical resection. Herein, a radical resection ought to be achieved, as a circumferential resection margin (CRM) of ≤ 1 mm increases the risk of local recurrence [5, 6]. To improve the chance of a clear CRM, preoperative radiation therapy as neoadjuvant treatment is standard of care in patients with a high risk for local recurrence, including patients with locally advanced rectal cancer (LARC) [4]. The addition of 5-fluorouracil (5-FU) to long course radiation therapy has shown to increase response rates [7, 8]. Disadvantages of continuous 5-FU infusion are the need of hospitalisation and complications related to central venous infusion. Both can lead to unwanted costs and a delay to surgery [8].

Capecitabine is an orally administered prodrug and can be used as an alternative for continuous 5-FU infusion as effective radiosensitizer during radiation [9, 10]. Although capecitabine may reduce practical difficulties compared to continuous 5-FU infusion, acute toxicity during preoperative chemoradiation still remains a problem [11]. The most common adverse effects of capecitabine are diarrhea and palmar-plantar erythrodysesthesia syndrome. Acute toxicity during chemoradiation with concomitant capecitabine, most commonly being severe diarrhea, could lead to an interruption or cessation of preoperative treatment and is potentially life-threating. Furthermore, dehydration and/or significant limitations to the patients’ self-care activities of daily living often results to the need of hospitalisation. Due to the great impact severe diarrhea has on both patient- and treatment outcomes, risk factors for should be identified and if possible corrected during the pre-treatment assessment.

Previous studies have identified low skeletal muscle mass as predictor for worse oncologic outcomes and toxicity during 5-FU based treatment in colorectal cancer patients [12–15]. However, the impact of body composition on toxicity during neoadjuvant chemoradiation with capecitabine has not yet been described. The objective of this study is to investigate possible risk factors for severe diarrhea in patients treated with neoadjuvant chemoradiation with capecitabine for rectal cancer.

## Methods

### Patients

All consecutive patients with LARC treated with concomitant chemoradiation with capecitabine in the Erasmus MC Cancer Institute from January 2009 until July 2015 were retrospectively reviewed. Patients with locally recurrent rectal cancer (LRRC) who received chemoradiation for the first time were also included. Patient information, pre-treatment tumour characteristics and toxicity were obtained through patients’ electronic medical records.

### Treatment

All patients were treated with radiation therapy combined with capecitabine. Radiation therapy consisted of a radiation dose of 50 Gy delivered in 25 fractions of 2.0 Gy over a period of five weeks. In addition, a flat dose of 1500 mg capecitabine orally twice daily was administered starting on the first day of radiation therapy till the last day of radiation therapy. Before the 1st of December 2011 patients were treated with capecitabine taken only on radiation days, that was given on weekdays. After this date, the treatment regime changed to capecitabine prescribed seven days a week during radiation therapy due to a change in the guideline. Treatment toxicity was evaluated during several outpatient visits by radiation therapists and medical oncologists. Dihydropyrimidine dehydrogenase (DPD) testing before the administration of capecitabine was not performed during the study period.

### Definitions

Estimated glomerular filtration rate (eGFR) was calculated by the CKD-EPI (Chronic Kidney Disease Epidemiology Collaboration) formula before the start of preoperative treatment. Decreased kidney function was defined as an eGFR of < 60 mL/min per 1.73 m^2^. Toxicity was scored according to the Common Terminology Criteria for Adverse events, version 4.0 (CTCAE v4.0). Herein, toxicity grade 3 was defined as either an increase of ≥ 7 stools per day over baseline, incontinence, hospitalization indicated, severe increase in ostomy output compared to baseline or limiting self-care activities of daily living (ADL). Toxicity events with grade 3 or higher were defined as severe diarrhea. Grade 1 and grade 2 diarrhea were defined as an increase of < 4 stools and an increase of 4–6 stools over baseline, respectively.

### Skeletal muscle mass assessment

**Fig. 1:**
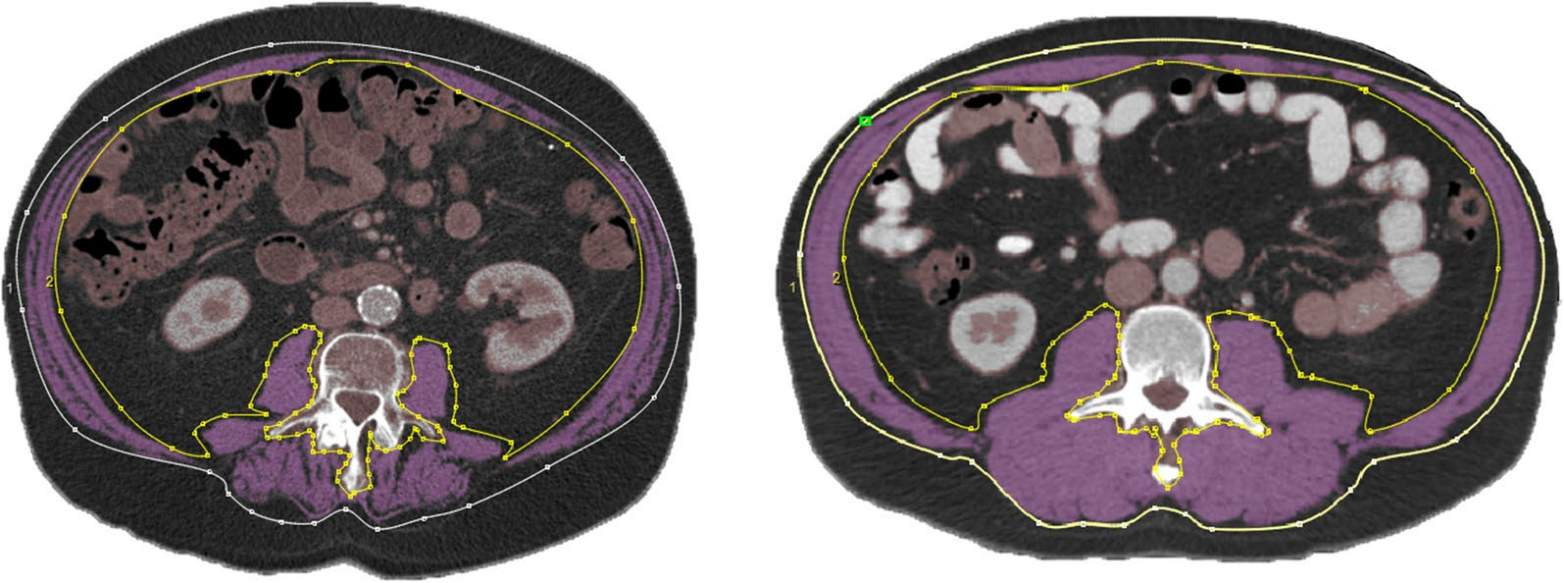
CT scans at the third lumbar vertebral level of a patient with sarcopenia (left) and a patient with normal skeletal muscle mass and density (right). The skeletal muscles are outlined

Skeletal muscle mass was estimated on standard, routinely performed pre-radiation computed tomography (CT) scans of the abdomen. The total cross-sectional skeletal muscle area was measured at the third lumbar vertebra (L3) and adjusted for patients’ body height squared to calculate the skeletal muscle index (SMI). International accepted cut off values described by Martin et al. were used to define low skeletal muscle mass [16]. Herein, low skeletal muscle mass was defined as SMI < 53 cm/m^2^ in male patients with body mass index (BMI) ≥ 25 kg/m^2^ and SMI < 43 cm/m^2^ in male patients with BMI < 25 kg/m^2^. Low skeletal muscle mass in female patients was defined as SMI < 41 cm/m^2^. In addition to skeletal muscle mass, skeletal muscle density at L3 was measured in Hounsfield units (HU). Low skeletal muscle density was defined as HU < 33 in patients with BMI ≥ 25 kg/m^2^ and HU < 41 in patients with BMI < 25 kg/m^2^. Muscle mass was measured with FatSeg, which is a validated developed software program to measure body composition on CT images [17]. An example of an abdominal CT scan at L3 level of a patient with sarcopenia and a patient with normal skeletal muscle mass and density is shown in Fig. 1.

### Statistics

Continuous data were reported as median with interquartile ranges (IQR) and categorical data were reported as counts (percentage). Missing data were not included in descriptive statics. Univariate logistic regression analyses were used to identify possible risk factors for severe diarrhea. Variables with a *p* value < 0.1 were included in the multivariable analysis. Multivariable logistic regression with backward selection was then used to identify the most statistically relevant predictors for severe diarrhea. Variables of interest were sex, age, BMI, T-stage, N-stage, M-stage, skeletal muscle density, skeletal muscle mass and renal function. In the multivariable regression model with backward selection the significance level was set at a *p* value < 0.05. Frequency distribution of severe diarrhea for patients with LRRC and patients who received continuous chemoradiation therapy (after the 1st of December 2011) were analysed separately as possible risk groups for severe diarrhea by Pearson's chi-squared test. Also, the occurrence of severe diarrhea was compared between female patients who underwent a hysterectomy in the past and female patients without a hysterectomy in the past. All analyses were performed using IBM Statistical Package for Social Sciences software (SPSS) version 25 and R version 3.6.1 (https://www.r-project.org/).

This study was approved by the medical ethics committee of the Erasmus MC (MEC-2016-262).

## Results

A total of 746 patients who received concomitant preoperative chemoradiation with capecitabine were included. Baseline characteristics and treatment details were summarized in Table 1. The median age was 64 years (IQR 57–71), 477 patients were male (64%) and 713 patients were treated for primary rectal cancer (96%). At baseline 325 patients had low skeletal muscle mass (51%), 278 patients had low skeletal muscle density (44%). Continuous dosing scheme of capecitabine was administered in 446 patients (60%). Decreased renal function was diagnosed in 51 patients (7%). In total, 70 patients (9%) experienced grade 3 to 5 diarrhea of whom 68 patients had grade 3, one patient had grade 4 and one patient had grade 5 diarrhea.

**Table 1:** Baseline characteristics and treatment details of rectal cancer patients treated with preoperative chemoradiation with capecitabine (n = 746)

Sex	
Male	477 (64%)
Female	269 (35%)
Age (years)	64 (57–71)
T-stage	
2	33 (5%)
3	539 (76%)
4	134 (19%)
N-stage	
0	102 (14%)
1	265 (37%)
2	346 (49%)
M-stage	
0	659 (90%)
1	74 (10%)
Primary rectal cancer	713 (96%)
Recurrent rectal cancer	33 (4%)
BMI (kg/m^2^)	25.8 (23.5–28.7)
Skeletal muscle mass	
Normal	306 (49%)
Low	324 (51%)
Skeletal muscle density	
Normal	353 (56%)
Low	278 (44%)
Capecitabine dosing scheme	
Weekdays only	300 (40%)
Continuous	446 (60%)
Renal function	
eGFR < 60 ml/min/1.73 m^2^	51 (7%)
eGFR ≥ 60 ml/min/1.73 m^2^	694 (93%)
Diarrhea	
No diarrhea	543 (73%)
Grade 1	90 (12%)
Grade 2	43 (6%)
Grade 3	68 (9%)
Grade 4	1 (0%)
Grade 5	1 (0%)

Percentages might not add up due to rounding

*BMI* body mass index, *eGFR* estimated glomerular filtration rate

### Logistic regression analyses

Results of logistic regression analyses were reported in Tables 2, 3 and 4. Risk factors which were associated with severe diarrhea in univariate logistic regression analysis were female sex (odds ratio (OR): 3.63, 95% confidence interval (CI) 2.19–6.15), age ≥ 65 (OR: 3.06, 95% CI 1.82–5.33), BMI (OR: 0.95, 95% CI 0.88–1.00), decreased kidney function (OR: 2.57, 95% CI 1.17–5.22) and low skeletal muscle mass (OR: 1.68, 95% CI 0.99–2.93). In the multivariable logistic regression analysis with backwards selection only female sex (OR: 4.42, 95% CI 2.54–7.91) and age ≥ 65 (OR: 3.25, 95% CI 1.85–5.87) remained associated with severe diarrhea.

**Table 2:** Univariate regression analyses for severe diarrhea

	OR (95% CI)	*p* value
Sex		
Male	Ref	
Female	3.63 (2.19–6.15)	< 0.001
Age		
< 65 years	Ref	
≥ 65 years	3.06 (1.82–5.33)	< 0.001
BMI (kg/m^2^)		
	0.95 (0.88–1.00)	0.073
T-stage		
T2	Ref	
T3	1.00 (0.34–4.28)	1.000
T4	1.07 (0.32–4.90)	0.915
N-stage		
N0	Ref	
N1	1.27 (0.60–2.94)	0.551
N2	0.87 (0.41–2.03)	0.739
M-stage		
M0	Ref	
M1	0.38 (0.09–1.06)	0.108
Skeletal muscle mass		
Normal	Ref	
Low	1.68 (0.99–2.93)	0.059
Skeletal muscle density		
Normal	Ref	
Low	1.21 (0.72–2.05)	0.470
Renal function		
eGFR < 60 ml/min/1.73 m^2^	Ref	
eGFR ≥ 60 ml/min/1.73 m^2^	2.57 (1.17–5.22)	0.012

*BMI* body mass index, *eGFR* estimated glomerular filtration rate, *Ref* reference

**Table 3:** Multivariable logistic regression analysis for severe diarrhea

	Adjusted OR (95% CI)	*p* value
Sex		
Male	Ref	
Female	4.41 (2.51–7.98)	< 0.001
Age		
< 65 years	Ref	
≥ 65 years	3.03 (1.70–5.55)	< 0.001
BMI (kg/m^2^)		
	0.95 (0.89–1.01)	0.132
Skeletal muscle		
Normal	Ref	
Low	1.16 (0.64–2.14)	0.632
Renal function		
eGFR < 60 ml/min/1.73 m^2^	Ref	
eGFR ≥ 60 ml/min/1.73 m^2^	2.07 (0.85–4.67)	0.090

*OR* odds ratio, *95% CI* 95% confidence interval, *BMI* body mass index, *eGFR* estimated glomerular filtration rate, *Ref* reference

**Table 4:** Multivariable logistic regression analyses (after backwards selection) for severe diarrhea

	Adjusted OR (95% CI)	*p* value
Sex		
Male	Ref	
Female	4.42 (2.54–7.91)	< 0.001
Age		
< 65 years	Ref	
≥ 65 years	3.25 (1.85–5.87)	< 0.001

*OR* odds ratio, *95% CI* 95% confidence interval, *Ref* reference

### Risk groups

Predesignated risk groups were analysed separately. One specific patient group that was especially at risk for severe diarrhea consisted of female patients with a hysterectomy before chemoradiation. The occurrence of severe diarrhea in 40 hysterectomy patients was significantly higher compared to 221 female patients who did not had this procedure in the past (n = 14 (35.0%) vs. n = 31 (14.1%), *p* = 0.003). Five of 38 patients with LRRC (15.6%) developed severe diarrhea compared to 64 of 713 (9.1%) patients with primary cancer (*p* = 0.391). Continuous dosing scheme of capecitabine was administered in 446 patients and no difference was found in the occurrence of severe diarrhea compared to the 300 patients treated with capecitabine on radiation days only (n = 47 (10.5%) vs. n = 23 (7.7%), *p* = 0.234).

## Discussion

In this retrospective single centre cohort study identifying risk factors for developing severe diarrhea during preoperative chemoradiation with capecitabine female patients and patients older than the age of sixty-five were most at risk for developing severe diarrhea (resp. unadjusted OR: 3.63, 95% CI 2.19–6.15 and 3.06, 95% CI 1.82–5.33). No relation between body composition and severe diarrhea was found after adjusting for sex, age and renal function.

Female sex was associated with an increased risk of severe diarrhea in both univariate and multivariable analysis, and females were over four times more likely to develop severe diarrhea than males. This finding is in line with previous studies reporting a both greater incidence as severity of toxicity in females treated with 5-FU based chemotherapy [15, 18, 19]. It is hypothesized that females experience more toxicity during 5-FU treatment due to variation in pharmacological metabolism such as levels of dihydropyrimidine dehydrogenase and thymidylate synthase [15, 20]. However, the prevalence of DPD deficiency is estimated to be only 0.1–2.8% in the whole population, and could therefore not explain the large proportion of patients (9.4%) experiencing severe diarrhea [21, 22]. In addition, neutropenia, that is commonly associated with DPD deficiency, was only found in 9 patients (1.2%) in our study. This suggests that other, considerably more important, factors have contributed to the increased occurrence of severe diarrhea in female patients.

Alternatively, differences in the pelvic anatomy between females and males may explain the higher rate of diarrhea in female patients. The fact that females have a larger and broader pelvis than males makes it likely that more small bowel volume is located in the pelvic area, and thus within the radiation field. It is well recognized that there is an important causal relation between the volume of small bowel irradiated and the development of diarrhea [23–25]. One finding in this present study that supports this hypothesis is that female patients with a hysterectomy in the past have a greater risk of developing diarrhea compared to female patients without hysterectomy (35.0% vs. 14.1%, *p* = 0.003). As more free space is left behind in the lower pelvic area after hysterectomy, it is plausible that more descended bowel is irradiated, hereby increasing the risk of receiving a toxic dosage. Although the dose–volume relationship between diarrhea and irradiated bowel volume is broadly established in literature, the role of female pelvic anatomy has not clearly been emphasised in these studies [23, 26–28]. Finally, differences between sexes in the experience of symptoms might also play a role in the (subjective) reporting of toxicity scores but fail to explain objective toxicity outcome measures such as the higher incidence of leukocytopenia among females found in other studies [15].

To investigate the impact of body composition, low skeletal muscle mass, low skeletal muscle density and BMI were analysed as possible risk factors for severe diarrhea. Low skeletal muscle mass and low BMI were both predictors for severe diarrhea, but this correlation was not statically significant after adjusting for sex, age and renal function. In further analysis, low skeletal muscle mass was significantly more common in female patients compared to male patients (61% vs. 47%, *p* = 0.003). The association between low skeletal muscle mass and severe diarrhea might therefore be confounded by sex. In the current study, patients with low skeletal muscle density had no increased risk for severe diarrhea compared to patients with normal skeletal muscle density.

The incidence of severe diarrhea in the current study is comparable to several other studies describing an incidence of 4.2–10.2% [9–11, 29–31]. In the study of Swellengrebel et al. patients were treated with continuous dosing of capecitabine and 10.2% patients developed severe diarrhea. The authors discussed the option of only prescribing capecitabine on days of radiation to optimize tolerability. In this study, patients treated before first of December 2011 received capecitabine only on radiation days, and patients after this date were treated with continuous dosing. Although statistical difference was not reached, the occurrence of severe diarrhea was more common in patients treated with continuous dosing compared to patients treated with capecitabine on radiation days (10.5% vs. 7.7%, *p* = 0.234).

In the current study, female patients and patients aged sixty-five or older were evidently more at risk for severe diarrhea. The remaining question of this research is how to translate these results into practice. One could argue that these specific patient groups should be offered an altered dosage of capecitabine or a different radiation scheme, for example short course radiation with a longer waiting period. A downsize of this strategy is the possibility of undertreatment of these patients, potentially resulting in less tumour downgrading and thus a higher risk of an irradical resection margin [4]. Another possible solution for diminishing toxicity rates during chemoradiation with capecitabine is the use prehabilitation programs. Promising results of the benefits of prehabilitation and exercise programs for rectal cancer patients undergoing chemoradiation treatment are emerging [32–34]. Targeting treatment on subgroups which have most advantage from it will eventually make prehabilitation programs more sufficient and cost-effective. However, whether prehabilitation actually reduces the risk of severe diarrhea in these patients is uncertain. Ongoing trials will hopefully give more insight in the optimisation of (personalized) prehabilitation for rectal cancer patients undergoing preoperative chemoradiation [33, 35, 36].

This retrospective cohort study from a single centre has several limitations. First, no dose–volume analyses of irradiated bowel were performed in this study. Secondly, DPD testing was not standard of care during the study period in the Netherlands and was therefore not conducted in our population. Patients with a (partial) DPD deficiency treated with 5-FU have an increased risk of developing toxicity [22]. Nowadays, prospective DPD screening and implicating DPD genotype-based dose reductions have resulted in a safer chemoradiation treatment regime [37]. Toxicity rates of chemoradiation in rectal cancer patients treated present day are therefore probably lower compared to patients in our population. It should also be acknowledged that presumed lower DPD activity in females may have contributed to the higher incidence of severe diarrhea in female patients found in this current study [38]. Another limitation of this study is the lack of follow-up data. Surgery and post-operative treatment was usually performed in referral hospitals. Important patient outcomes such as surgical complications and long term oncologic survival were therefore not investigated.

In conclusion, this study demonstrates that female patients and patients aged sixty-five or older are especially at risk for severe diarrhea during preoperative chemoradiation therapy with capecitabine. Due to the retrospective nature of this study, no comprehensive explanation for the higher toxicities rates among these patients could be determined. These findings however suggest that high risk patients should be treated with caution and that alternative neoadjuvant treatment methods might be considered. In the future, high risk patients could, for example, be followed-up more frequently, scheduled with treatment breaks or administered with an adjusted dosage of radiosensitizer (e.g. capecitabine).

## Data Availability

The datasets used and/or analysed during the current study are available from the corresponding author on reasonable request.

## Abbreviations

5-FU: 5-Fluorouracil
ADL: Activities of daily living
BMI: Body mass index
CI: Confidence interval
CKD-EPI: Chronic Kidney Disease Epidemiology Collaboration
CRM: Circumferential resection margin
DPD: Dihydropyrimidine dehydrogenase
eGFR: Estimated glomerular filtration rate
HU: Hounsfield units
L3: Third lumbar vertebra
LARC: Locally advanced rectal cancer
LRRC: Locally recurrent rectal cancer
OR: Odds ratio
SMI: Skeletal muscle index
